# Hollow-Core Photonic Crystal Fiber Mach–Zehnder Interferometer for Gas Sensing †

**DOI:** 10.3390/s20102807

**Published:** 2020-05-15

**Authors:** Kaveh Nazeri, Farid Ahmed, Vahid Ahsani, Hang-Eun Joe, Colin Bradley, Ehsan Toyserkani, Martin B. G. Jun

**Affiliations:** 1 Department of Mechanical Engineering, University of Victoria, Victoria, BC V8W 2Y2, Canada; nazerik@uvic.ca (K.N.); ahsaniv@uvic.ca (V.A.); cbr@uvic.ca (C.B.); 2 Department of Mechanical and Mechatronics Engineering, University of Waterloo, Waterloo, ON N2L 3G1, Canada; ehsan.toyserkani@uwaterloo.ca; 3 School of Mechanical Engineering, Purdue University, West Lafayette, IN 47907, USA; hjoe@purdue.edu (H.-E.J.); mbgjun@purdue.edu (M.B.G.J.)

**Keywords:** refractive index sensor, gas sensor, hollow-core photonic crystal fiber, Mach–Zehnder interferometer

## Abstract

A novel and compact interferometric refractive index (RI) point sensor is developed using hollow-core photonic crystal fiber (HC-PCF) and experimentally demonstrated for high sensitivity detection and measurement of pure gases. To construct the device, the sensing element fiber (HC-PCF) was placed between two single-mode fibers with airgaps at each side. Great measurement repeatability was shown in the cyclic test for the detection of various gases. The RI sensitivity of 4629 nm/RIU was demonstrated in the RI range of 1.0000347–1.000436 for the sensor with an HC-PCF length of 3.3 mm. The sensitivity of the proposed Mach–Zehnder interferometer (MZI) sensor increases when the length of the sensing element decreases. It is shown that response and recovery times of the proposed sensor inversely change with the length of HC-PCF. Besides, spatial frequency analysis for a wide range of air-gaps revealed information on the number and power distribution of modes. It is shown that the power is mainly carried by two dominant modes in the proposed structure. The proposed sensors have the potential to improve current technology’s ability to detect and quantify pure gases.

## 1. Introduction

Gas sensing is essential for safety and maintenance operations in many industries, including the power generation [1], petrochemical [2], and food-processing sectors [3]. For detecting the presence of gases, especially in extreme conditions, the silica optical fiber provides a promising platform, due to its unique properties. These include immunity to electromagnetic radiation [4], high-temperature durability [5], compactness, as well as high accuracy and sensitivity [6]. Researchers have pursued the applicability of optical fiber sensors across many sensing applications, because of their multifunctional sensing capabilities (e.g., refractive index (RI), temperature, and pressure) [7]. The various mechanisms that have been investigated for gas-sensing functionality include Raman scattering [8], surface Plasmon resonance [9], evanescent-field absorption [10], derivative spectroscopy [11], and interferometric sensors [6]. Successes in these research projects relied upon experimentation with a range of optical fibers: D-shaped fiber, multimode fiber, fused silica fiber optic bundles, and photonic crystal fiber (PCF) [6,8,9,10,11]. Various types of fiber optic interferometers have been studied for their RI-sensing capabilities: the Sagnac, Michelson, Fabry–Perot, and Mach–Zehnder interferometers (MZIs) [6]. Wang et al. [12] developed a micro Fabry–Perot cavity interferometer and achieved the RI sensitivity of 851 nm/RIU while having a very low-temperature sensitivity of 0.27 pm/°C and low-temperature cross-sensitivity of 3.2 E^−7^ RIU/°C. Hu et al. [13] proposed an intrinsic Fabry–Perot interferometer based on simplified hollow-core fiber and achieved a RI measurement resolution of 6.5 E^−5^. These types of sensors typically show low insertion loss and they are relatively easy to fabricate. A Michelson interferometer was constructed by splicing a stub of large-mode-area PCF to single-mode fiber (SMF) and an RI resolution of E^−4^ in the RI range of 1.33–1.45 was reported [14]. Facile fabrication procedure and high stability over time were reported as key advantages. Sun et al. [15] proposed a hybrid interferometer by forming a Fabry–Perot cavity in one of the optical paths of the Michelson interferometer. The spectral response of this hybrid sensor allows multiparameter sensing as it has two distinct interference fringes. The simultaneous measurement capability was reported with an RI measurement resolution of 8.7 E^−4^ in the RI range of 1.33–1.38 with a temperature sensitivity of 13 pm/°C. A photonic crystal fiber Sagnac interferometer was developed by Liu et al. [16] as an RI sensor, by filling the central hole of the fiber with microfluidic analytes. Fabrication of these sensor types are complicated as filling air holes of a PCF is challenging. A high sensitivity of about 19,000 nm/RIU with a resolution of 1.05 E^−6^ was achieved in their work. MZI based optical sensors have received significant attention because they are robust, compact [17], and low-cost units that also have high levels of precision [18]. 

Researchers have proposed disparate configurations in fabricating in-line MZI sensors for sensing ambient RI changes. Implementation techniques already tested extend from core mismatch splicing of optical fibers [19] to cladding collapse of PCF [20], tapering of fibers [21], the use of microfiber [22], and splicing of hollow-core fiber [23]. Similarly, many approaches have been used in attempts to enhance ambient refractive index sensitivity of fiber-optic MZIs. Huang et al. [18] developed a thin-core fiber-based MZI for ammonia sensing with a sensitivity of 850 nm/RIU in the RI range of 1.5–1.518. In other studies, graphene-coated fiber-optic MZI sensors were found to have gas sensing sensitivity in the range of 3–6 pm/ppm [24,25]. Duan et al. [26] engineered a compact MZI by creating a short-length (62.5 µm) of cavity through offset-splicing the SMFs on both ends. Their innovative design resulted in a sensitivity of 3400 nm/RIU in the RI-range of 1.0 to 1.0022. PCF has also proven to be an excellent choice for fabricating RI sensors because the effective RI of the propagating cladding mode is highly sensitive to the surrounding environment [27,28,29]. Yang et al. [29] demonstrated the viability of a compact PCF Mach–Zehnder refractometer for sensing methane. They coated a polymer (fluoro-siloxane) over the internal surface of air holes, with one end of the PCF fusion spliced to an SMF while the other end was open for gas-molecule penetration. Through this fabrication technique, a sensitivity (defined as wavelength change per percentage of methane) of 0.514 nm%–1 was achieved [29]. This otherwise promising sensor type has drawbacks; it requires a long response time when retrieving initial conditions and also has a low level of gas selectivity.

The article by Cregan et al. in 1999 was the first research that utilized HC-PCF for the application of gas detection [30]. The presence of hollow channels in a fiber’s core and cladding regions makes it difficult to fusion splice an HC-PCF to an SMF. The air holes in HC-PCF hold a large volume of air. During fusion splicing, air will expand and distort the fiber structure. In 2011, Qu et al. [31] suggested using hollow-core fiber to infiltrate various aqueous analytes in high RI measurements with a sensitivity of 1400 nm/RIU. Subsequent to this innovative proposal, a 5.1 m HC-PCF gas cell was used for the detection of methane [32]. Generally, it takes time for gas molecules to fill the cavities of HC-PCF, so this technique makes a delay in the initial measurement response to the presence of the gas [33]. Furthermore, Wynne et al.’s [34] suggestion regarding the pressure-driven filling of air-holes with gases is not applicable for real-time monitoring. Moreover, focused ion beam or femtosecond laser-assisted micro-channels can be fabricated on the cladding of HC-PCF to accelerate gas diffusion [35,36]. Nicholas et al. [37] proposed an HC-PCF-based MZI using ceramic ferrules to connect a 344-mm-long HC-PCF to two SMFs. An alternative HC-PCF-based MZI gas sensor has been reported, which employs the HC-PCF as one of the interferometer’s arms [38]. Many of the sensors proposed to date either have complex configurations or poor sensitivity and response time for high-resolution measurement of gases. Ahmed et al. [39] reported a highly sensitive MZI structure that uses a small stub of HC-PCF for monitoring of CO_2_; however, a detailed study on such a configuration is necessary to better understand its performances and to explore other potential applications. Recently, we studied length-dependent performance of these devices to understand their sensing properties [40]. However, more studies are required to better understand design parameters and sensing performance of these MZI sensors.

An in-line fiber optic MZI sensor, which is compact and robust with high sensitivity, is presented in this report. The HC-PCF MZI sensor utilized a short length of HC-PCF placed in between two SMFs, with gaps at each interface. The light propagation, working principles, and essential performance parameters of the proposed gas sensor are presented in this study. These include response and recovery times, RI sensitivity, as well as the number and power distribution of modes. Relative RI detection was used in all experiments, because of the difficulties in absolute RI measurement with high accuracy [41]. Experiments show promising results in the sensor’s RI sensitivity. The device responds well to different gases and shows good repeatability on gas detection. 

## 2. Working Principles

**Figure 1:**
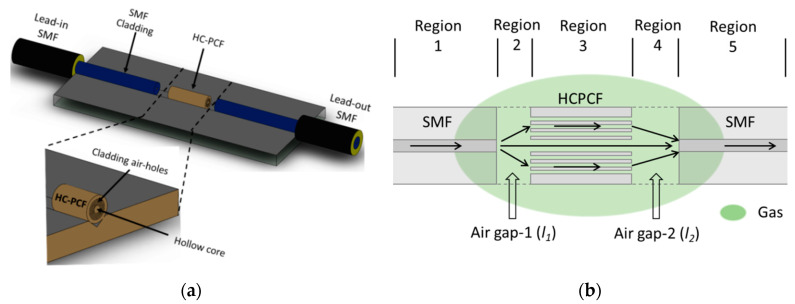

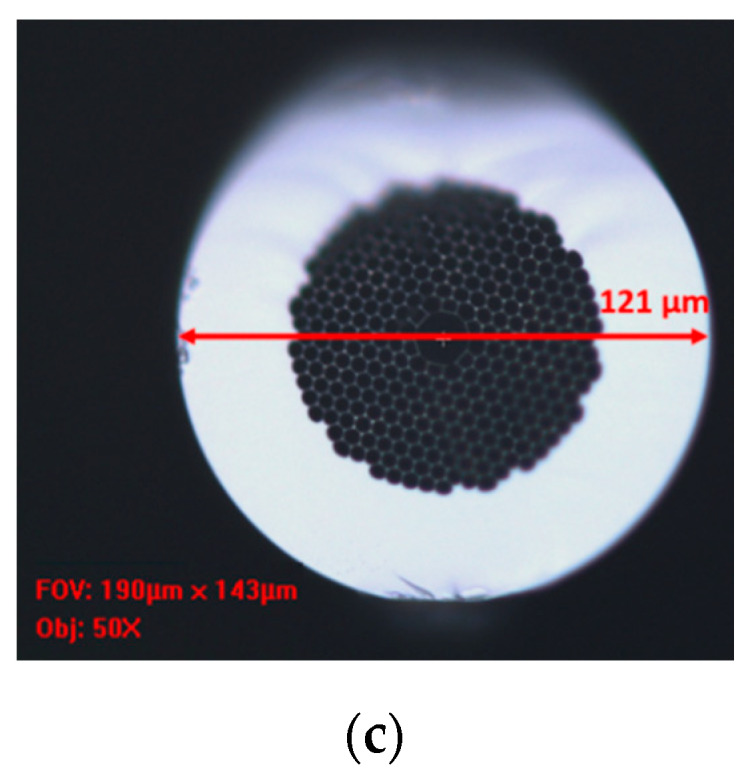
(**a**) Schematic of the proposed sensor arrangement, (**b**) schematic of light transmission within the sensor, and (**c**) microscopic image of the cross-section of 10-micron hollow-core photonic crystal fiber (HC-PCF) fiber. SMF = single-mode fiber.

Figure 1a schematically shows a fiber arrangement of the proposed MZI sensor. A short length of HC-PCF was positioned on the V-groove and aligned with SMFs. There is an air gap at each end of the sensing element fiber. The schematic illustration of light transmission in the sensor is shown in Figure 1b. The lead-in SMF carries the incoming light wave. It radiates from the SMF core after reaching the first sensor gap in region 2 and acts as a pseudo-point light source. In the first air gap, the fundamental mode broadens and when it reaches the HC-PCF both fundamental and higher-order modes are excited in the circular channels of the sensing element. Interaction between the light and the gas molecules takes place in region 3 along the length of the sensor. Optical interference occurs in region 4 (second gap) due to the phase difference between the fundamental mode and higher-order modes. The lead-out SMF then transfers the interference spectrum to an interrogator (or spectrum analyzer). The device’s reference and sensing arms are both in contact with gas molecules; however, the effect of RI change on the interference in the sensing arm is higher than in the reference arm. That imbalance occurs due to differences in optical-path lengths and phase shifts between the arms.

Figure 1c shows the cross-section of the HC-PCF used in this study. This fiber offers low index guiding of light as the core-index of the HC-PCF is lower than the effective index of the cladding [42]. The photonic bandgap effect makes propagation impossible in the microstructure cladding leading to light confinement in the core. This design enhances gas sensing capabilities as the HC-PCF provides a remarkably strong interaction between gas molecules and light particles, due to strong field confinement [43,44]. Higher-order core modes and surface modes are supported by HC-PCF fibers [45]. The optical path difference between the reference arm and sensing arm defines the fiber-optic MZI sensor’s interference spectrum. Such interference is a function of core intensity (I core), cladding intensity (I cladding), and phase difference (ϕ) [17,46], which can be written by the following equation:

(1) $$I=I_{\mathrm{core}}+I_{\mathrm{cladding}}+2\sqrt{I_{\mathrm{core}}I_{\mathrm{cladding}}}\cos\emptyset$$

Modes that are traveling the same distance (L) will have the phase difference (Δϕ) of:

(2) $$\Delta \phi =2\pi \left(\Delta n_{\mathrm{eff}}\right){L\;\lambda }^{-1}$$

Δn_eff_ is the difference in the effective RI between the core and cladding modes in equation 2, λ is the input wavelength, and L is the length of the HC-PCF path. Maximum transmission occurs at ΔΦ = 2πm (m is an integer) and peaks forms on the transmission signal at the following wavelengths:

(3) $$\lambda_{m}=\left(\Delta n_{\mathrm{eff}}\right){L\;m}^{-1}$$

Therefore, the mth order spectral shift can be written as:

(4) $${\Delta \lambda }_{m}=\left({\Delta n}_{\mathrm{eff}}+\Delta n\right){L\;m}^{-1}-{\Delta n}_{\mathrm{eff}}{\;m}^{-1}={\Delta \mathrm{nL}\;m}^{-1}$$

L is constant in the above equation and consequently, a change in the refractive index of the MZI’s core and cladding will change Δn and correspondingly ${\Delta \lambda }_{m}$. Consequently, a shift occurs at the transmission spectrum of the device and such change can be used for sensing of a measurand. 

## 3. Experimental Procedures

### 3.1. Fabrication of the MZI Sensor

**Figure 2:**
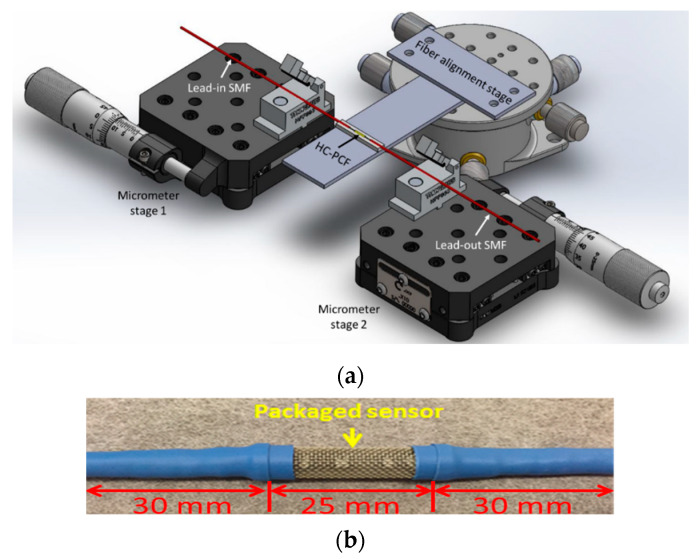
(**a**) Isometric view of the fabrication setup using two linear-translation micro stages for accurate control of gap distances, (**b**) packaged sensor using meshed stainless steel tube.

Two types of fibers were used to fabricate the HC-PCF MZI sensors: the SMF (Corning SMF28) and the HC-PCF (NKT Photonics HC-PCF 1550). Lead-in and lead-out fibers are standard single-mode fibers (SMF-28) with a core diameter of 8.2 µm, numerical aperture of 0.13, and a mode field diameter (MFD) of 9.3 µm (±0.5 µm). This sensor type utilizes an NKT Photonics HC-PCF fiber (HC-PCF 1550) as the sensing element. The HC-PCF fiber has a numerical aperture (NA) of 0.2, MFD of 9.00 µm (±1 µm) and core diameter of 10.00 µm. This sensing fiber element also has cladding air holes of diameter 3.10 µm and a cladding pitch of 3.80 µm. These fibers can guide several modes within a transmission of 1490 to 1680 nm [30]. In constructing the sensor, the SMFs and HC-PCF were assembled on a standard microscope glass slide (25 mm × 5 mm × 1 mm). Micro-machining created a V-groove on the microscope glass (25 mm length, 95 μm width, and 48 µm depth) using a femtosecond laser, which is used to align fibers. A CT-30 Fujikura cleaver was used to cleave fibers. To be able to cleave short lengths of HC-PCF in the order of a few millimeters, it was necessary to extend the length of the adapter plate to decrease the distance between the cutting blade and the adapter plate. Therefore, a 4 mm long aluminum plate was machined and marks at increments of 1 mm on it. Attaching the extension plate to the adapter plate made it possible to cleave fibers with lengths down to 2 mm. The cleaved stub of HC-PCF was positioned in the middle of the V-groove and fixed using epoxy glue. The exact length of the fiber, as well as the cleaving angles on both sides of the cleaved HC-PCF, were checked by examining them under an optical tooling microscope. Afterward, the single-mode fibers were positioned in fiber holders mounted on linear-translation micro stages and aligned with the sensing element fiber on the V-groove. Figure 2a shows an isometric view of the fabrication setup. To achieve a strong interference spectrum, gap lengths on both sides of HC-PCF were accurately adjusted. In this way mode splitting and recombination can be controlled. Fibers were then glued to microscope glass when an acceptable signal was observed. To provide mechanical strength to the assembly, the glass slide was secured in a meshed stainless steel tube, as shown in Figure 2b. Testing proved the robust effectiveness of the resulting sensor. Spacing between the HC-PCF and SMFs enabled ambient gas to diffuse into the HC-PCF air holes. 

**Figure 3:**
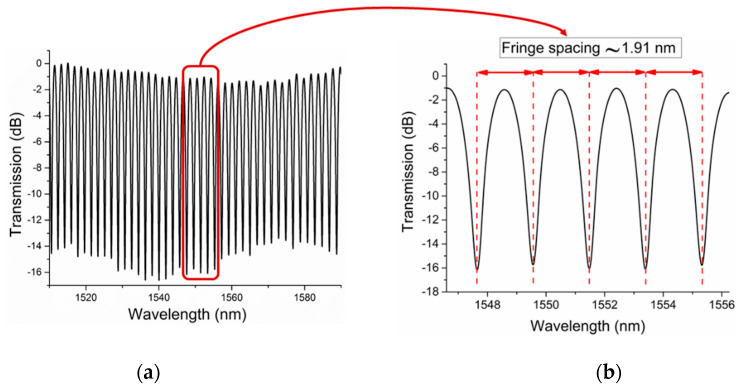
(**a**) Normalized transmission spectrum of an HC-PCF Mach–Zehnder interferometer (MZI) sensor with HC-PCF length of 3.3 mm and gaps of 1mm immersed in Nitrogen at room temperature and atmospheric pressure, (**b**) fringe spacing of the same sensor.

The normalized transmission spectrum of a sensor with an HC-PCF length of 3.30 mm and a gap distance of 1 mm on each side (Sensor C) is shown in Figure 3a. Figure 3b shows the fringe spacing of the same sensor. The measurement was taken when the device was immersed in Nitrogen (99.99% pure, atmospheric pressure) at room temperature. Each valley measured at the sensor’s output, see Figure 3a, results from interference between the signal arms in the MZI at that wavelength. The magnified spectrum graph shows a fringe spacing of 1.91 nm and a full width at half maximum (for transmission dip) of 0.47 nm. For the same configurations, the fringe spacings of sensor A (L = 4.97 mm) and sensor B (L = 4.73 mm) are 1.70 nm and 1.74 nm, respectively. The fringe spacing of the transmission spectrum increases as the length of HC-PCF decreases.

### 3.2. Spatial Frequency Analysis

In order to analyze the modes participating in the modal interference process, the transmission spectrum of MZIs with 4 mm of HC-PCF as a sensing element was Fourier transformed. This process allowed us to obtain the sensor’s corresponding spatial frequency, described as $v=\frac{\Delta n_{\mathrm{eff}}.D}{\lambda^{2}}$ [47], where $\Delta n_{\mathrm{eff}}$ represents the effective RI-difference between core and cladding of the sensing element and D is the distance between SMFs at each of the sensor’s ends. D varies from 4 mm to 16 mm in 500-micron increments. Different peaks in the spatial frequency graph correspond to the interference between the fundamental mode and different higher-order modes.

**Figure 4:**
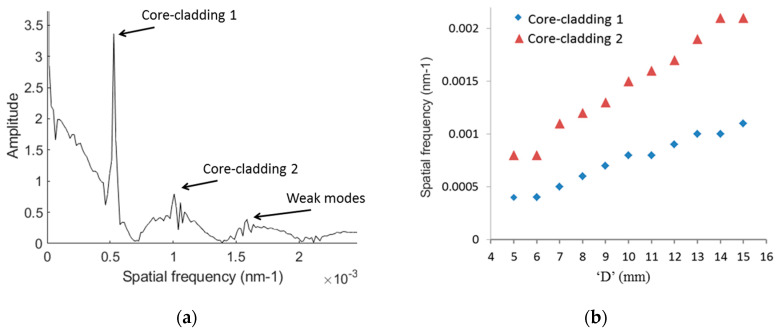
(**a**) Spatial frequency graph for MZI with HC-PCF length of 4 mm and D of 7 mm, (**b**) tracking dominant modes of the sensor for D. D = length of the sensing element.

Testing the MZIs with 10 µm HC-PCF as their sensing element revealed several multimodal-interference patterns occurring in the transmission spectrum. Further, in such a sensor, power is mainly distributed between two dominant modes in the spatial frequency spectrum, a finding that holds true across the entire range of gap distances. This phenomenon confirms that higher-order modes would gradually leak off the sensing fiber, contributing to transmission losses. So, fewer peaks would turn up in the spatial frequency graph due to a weakening interference-effect. As an example of the described effect, Figure 4a presents the spatial frequency graph for an MZI with 4 mm of 10 µm HC-PCF and gaps of 1.5 mm on each side (D = 7 mm). The sensor has a strong cladding mode with a spatial frequency of 5 × 10^−4^ (1/nm) and a normalized fast Fourier transform (FFT) value of 3.14: labeled core-cladding 1. Besides this dominant cladding mode, the sensor has a relatively weaker cladding mode (core-cladding 2) with a spatial frequency of 1.1 × 10^−3^ (1/nm) and a normalized FFT value of 0.99. Experimental findings show that for gaps from 0 to 1.65 mm, core-cladding 1 is the dominant cladding mode, while for higher gaps core-cladding 2 became the dominant mode. The highest power transmission resulted in MZIs with gaps of 1.35 mm, and the amplitude of spatial frequencies was seen to decrease intensely for gaps greater than 4.5 mm. Figure 4b was plotted by tracking dominant modes to show how the magnitude of spatial frequencies increases by increasing gap lengths for this sensor.

### 3.3. Characterization

**Figure 5:**
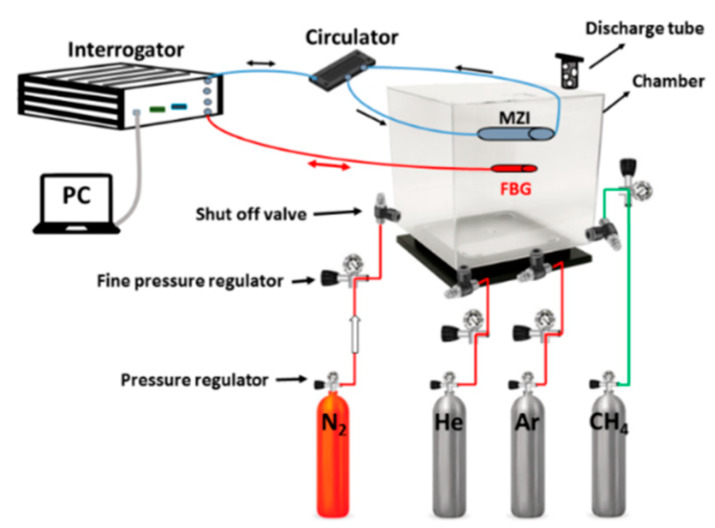
Schematic of the experimental setup; tests were carried out at atmospheric pressure and room temperature.

In the first set of experiments, RI measurements using three MZIs (constructed with different lengths of HC-PCF as their sensing elements) were carried out and their relative performances were compared. Figure 5 schematically shows the sensor evaluation system that includes the optical interrogator, a circulator, the MZI sensor, a Fiber Bragg Grating (FBG), reference gas tank, and measurand gas tanks. The MZI sensors under investigation were placed in a chamber with four gas intake valves. Reference nitrogen (N_2_), and measurand gas-tanks (‘He’, ‘Ar’, and ‘CH_4_’) were connected to these valves. The experiment used helium, methane, and argon with purity levels of 99.999%, 99%, and 99.99%, respectively. Using pressure regulators, an injection pressure of 15 psi was maintained during the testing process. To maintain constant pressure in the test chamber a discharge tube with a bubbler was connected to the test chamber. An interrogator (SM125) with a resolution of 1 pm was used to record and evaluate changes in the transmission spectrum. In addition, a FBG (sensitivity ~10 pm/°C) was positioned in the chamber to monitor and record the temperature variations. The spectral shifts of three sensor types and FBG were analyzed using the Micron Optics’ Enlight software. The experiments started with injecting N_2_ into the test chamber for long enough time to make sure an even gas diffusion into the air holes of HC-PCF was achieved. Measurand gases were then injected into the chamber (‘He’, ‘Ar’, or ‘CH_4_’). Using the mentioned software, spectral responses were recorded. Response and recovery times as well as refractive index sensitivity are among important sensing performance parameters of a gas sensor and were studied for three MZIs. The cyclic tests were performed using the various sensors to inspect the repeatability of RI measurements. Temperature, pressure and the injected gas species determined the spectral response of each sensor. Therefore, MZI sensors were temperature-characterized to compensate for the effect of temperature fluctuations during the experiments.

Another set of experiments sought to analyze the effect of gap distances on modal interference in the proposed MZI gas sensor. Here, lead-in and lead-out SMFs were not glued to the glass to facilitate easy adjustment of both airgaps. Using linear micro stages, gap lengths increased from 0 to 6 mm in 500-micron increments. Ensuring equal gap distance on both sides, we collected transmission spectrums for an interferometer with 10 μm HC-PCF as its sensing fiber. Spectrums were Fourier transformed to produce spatial frequency graphs, to explore the power distribution and the number of the sensor’s modes.

## 4. Results and Discussion

### 4.1. Refractive Index Sensing

**Figure 6:**
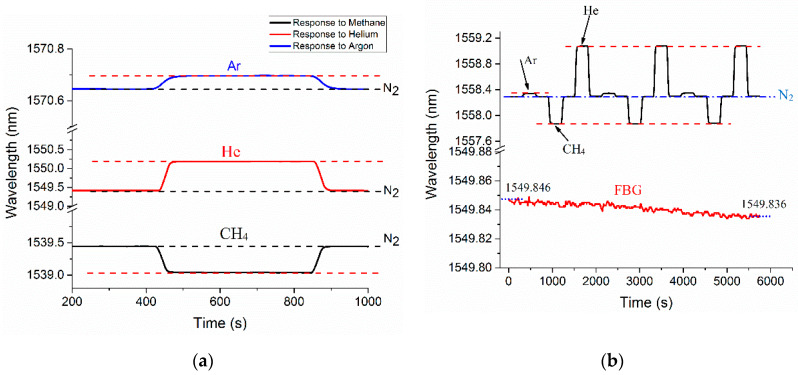
(**a**) The spectral shifts of sensor A when immersed in argon, helium, and methane injections, (**b**) the results of sequential sensing of measurand gases with sensor A, with gas injections carried out in the sequence of argon, methane, and helium.

Figure 6a illustrates the responses of sensor A (L = 4.97 mm) to methane, argon, and helium for one cycle. MZI sensors were exposed to measurand gases separately, to determine its spectral response to each gas. The sensor was interrogated with each measurand gas to investigate its spectral response in a complete test cycle. Each cycle started with the injection of Nitrogen (99.99% pure) until saturation followed by injection and measurement of target gas; and finally, an injection of Nitrogen back into the chamber, to purge the gas. The injection of gases was carried out for 7 minutes at each stage of a test cycle. As shown in Figure 6, the ambient gas in the test chamber determines the sensor’s wavelength response. Considering the location of the spectrum in N_2_ as the reference, sensor A showed spectrum shifts of 780 pm (±6 pm) when immersed in helium, 45 pm (±1 pm) when immersed in argon, and 440 pm (± 3 pm) when immersed in CH_4_. Spectral shifts of three valleys at different wavelengths were used to estimate mean wavelength shifts and measurement errors. Redshifts were recorded in the transmission spectrum for Ar or He and blue shifts were recorded for CH_4_. This finding can be explained in terms of spectral response to RI change. For a given ambient RI, the sensor’s transmission spectrum shows redshift to a negative RI change and blue shift to a positive RI change. In standard conditions, the RI values of He, Ar, N_2,_ and CH_4_ are 1.0000347, 1.0002820, 1.0002944, and 1.0004365, respectively. The interference fringe showed a redshift in the presence of helium and argon because RI of nitrogen is higher than their RIs. In contrast, the spectrum underwent a blue shift for methane, as the RI of nitrogen is lower than the RI of methane. The transmission fringe shifts of MZI sensors for helium, methane, and argon are listed in Table 1.

Sensor C, which has the shortest length of HC-PCF, shows the highest wavelength shifts among the three sensors tested when interrogated with all three gases. In contrast, sensor A, which has the longest HC-PCF stub of the three sensors, shows the smallest shifts. The RI sensitivities of the interferometric sensors are listed in Table 1, all falling in the RI range of 1.0000347–1.0004365. This RI range was selected based on the availability of gas tanks, and it could be extended in future research. The highest sensitivity was achieved by sensor C: 4629 (nm/RIU). This suggests that the RI sensitivity of the HC-PCF MZI sensors increases as the length of the HC-PCF stub decreases. As the next step in our experiments, argon, methane, and helium gases were sequentially injected into the test chamber, to investigate the sensors’ capacities to detect multiple gases. In each test cycle, the gas injection was carried out in the sequence of N_2_, Ar, N_2_, CH_4_, N_2_, He, and N_2_. This sequence was then repeated three times to determine sensing repeatability. Figure 6b shows the sequential gas response for sensor A, where the test cycles produced identical results. An FBG was used to record any temperature variation during the test. A maximum temperature fluctuation of 1 °C was recorded during the entire experiment. 

**Figure 7:**
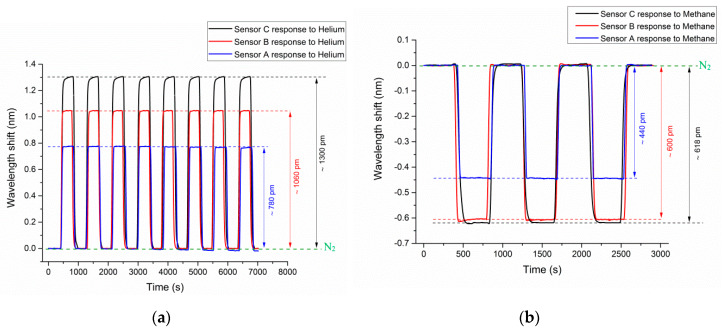
The normalized cyclic response of HC-PCF MZI sensors to (**a**) helium and (**b**) methane.

To check the consistency of the sensor’s measurements, repeatability tests were performed using all three sensors. For each test cycle, the sequential injection of nitrogen, measurand gas, and nitrogen was performed at 5 minutes intervals. Figure 7a shows the repeatability of sensing helium gas using all three sensors for eight cycles. The repeatability test for sensing methane gas was conducted for three test-cycles, as shown in Figure 7b. Both graphs below show the normalized wavelength shift that resulted when the chamber was sequentially filled with nitrogen and measurand gases. The data shows great repeatability of gas detection using the proposed HC-PCF interferometer.

**Figure 8:**
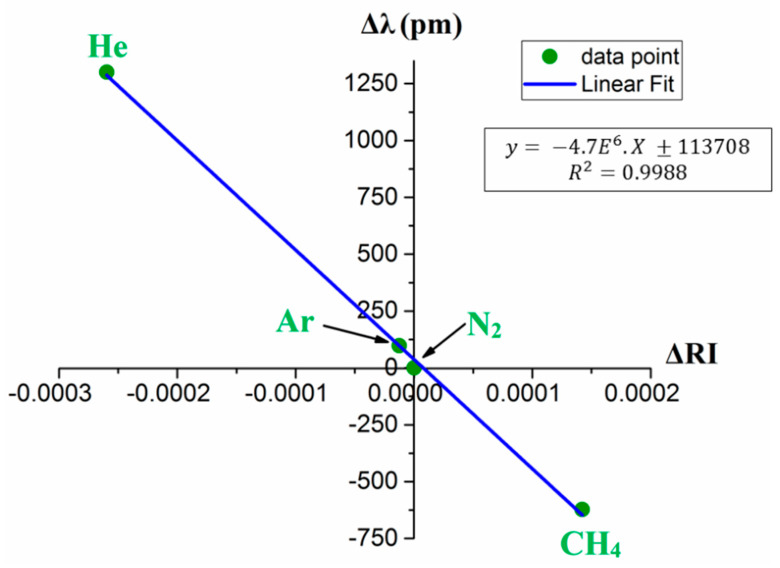
Sensitivity graph for sensor C to ambient RI change.

The RI sensitivity of sensors A, B, and C are 3019 nm/RIU, 4300 nm/RIU, and 4629 nm/RIU, respectively. Figure 8 shows the RI sensitivity of sensor A in the mentioned RI range. These data points were obtained via five separate measurements with a measurement error of ± 1 E^−6^, ± 2.3 E^−6^, and ± 5 E^−7^ for methane, helium, and argon, respectively. The proposed sensor configuration can improve on current technology, due to its linear RI response and high sensitivity to gases. The proposed interferometric sensor has, nonetheless, the potential for advancing current capacity for gas detection, quantitatively analyzing changes in pure gases as well as environmental monitoring applications. The RI characterization tests were conducted using an optical interrogator that has a wavelength accuracy of 1 pm (0.001 nm). Therefore, the sensor C (sensitivity of 4629 nm/RIU) has a RI resolution of 2.1 E^−7^. Similarly, the sensing resolution of sensors A and B can be calculated. 

The refractive index of any target gas (RI _target gas_) can be written as:

(5) $${\mathrm{RI}}_{\mathrm{target}\;\mathrm{gas}}={\mathrm{RI}}_{N_{2}}-\Delta \mathrm{RI}={\mathrm{RI}}_{N_{2}}-\Delta \lambda /\left(\mathrm{RI}\;\mathrm{sensitivity}\right)$$

The spectral shift, Δλ in the above equation can be attained by tracking valleys of transmission fringe of a sensor, as shown in Figure 6 and Figure 7. $RI_{N_{2}}$ is the refractive index of nitrogen, and ΔRI is the relative difference in RI between nitrogen and measurand gas. By knowing the wavelength shift (Δλ) and sensitivity of the MZI sensor, ΔRI can be calculated. 

Table 2 compares the sensitivity achieved in the present research with other similar and alternative studies available in published works. The table shows that the proposed MZI configuration shows much higher sensitivity in gas sensing compared to its counterparts in the RI range of 1 to 1.02. As shown in [40], decreasing the length of HC-PCF, the sensitivity of this sensor can be further improved. The proposed sensor is fairly compact (3.3 mm) compared to other HC-PCF based RI sensors [31,32,37,38], some of which are as long as ~35 cm. Therefore, the proposed MZI configuration is believed to perform much better in single-point gas sensing. It is worth mentioning here that even though a compact Fabry–Perot fiber sensor (in the range of micrometer) can be fabricated using ultrafast laser micromachining they have relatively poor RI sensitivity [12]. Despite its excellent gas sensing capabilities, the reported device has few drawbacks including fabrication complexity as it requires alignment and positing of the HC-PCF stub and cross-sensitivity to other measurands such as temperature and pressure. With the recent improvement in automated fiber alignment and positing systems, we believe the fabrication complexity can be drastically reduced for commercial applications. Similar to other fiber-optic sensors, the cross-sensitivities can be eliminated or reduced using an in-line fiber sensor such as a properly packaged FBG. The demonstrated sensor also needs to be packaged with a suitable membrane for selective sensing of gasses.

### 4.2. Sensor Response and Recovery Times

**Figure 9:**
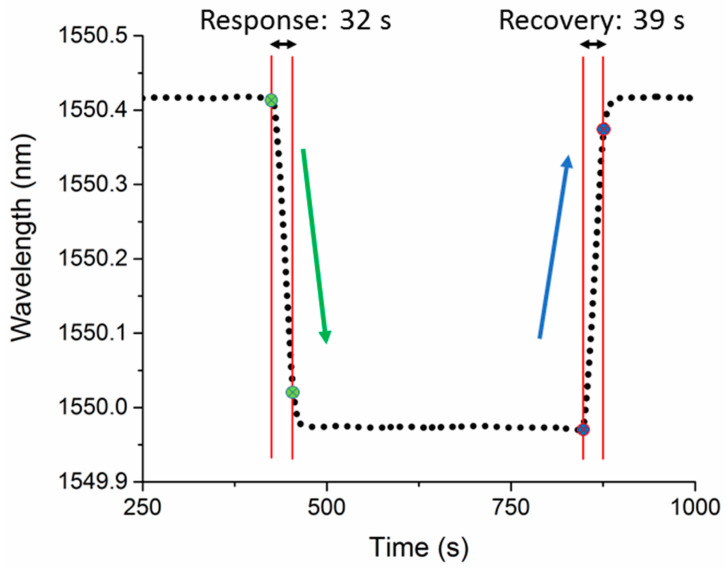
Response and recovery times of sensor A for methane.

Figure 9 illustrates the response and recovery times of sensor A for one cycle of methane sensing. The time duration that an MZI device takes to reach 90% of the total wavelength shifts is defined as response/recovery times. Accordingly, response and recovery times of sensor A are 32 s and 39 s for methane. Response and recovery times of three HC-PCF MZI sensors to methane, helium, and argon are listed in Table 3. Each reported time in this table is an average of five response or recovery times. Results indicate that sensor A, which has the longest HC-PCF stub, shows the fastest response/recovery times. However, the highest RI sensitivity was achieved using sensor C, which has the shortest length of HC-PCF. Response and recovery times depend on HC-PCF lengths and the volume of the test chamber. The test chamber has a dimension of 14.5 cm × 11.2 cm × 4.4 cm. 

### 4.3. Temperature Characterization

**Figure 10:**
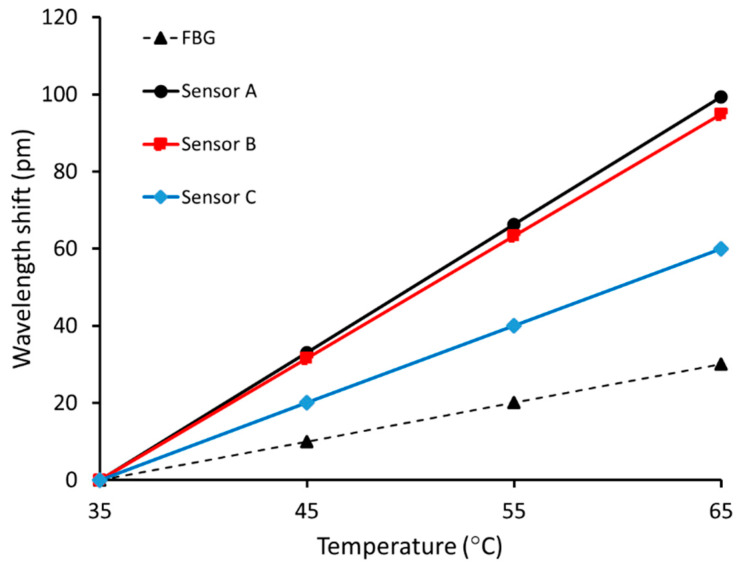
Temperature characterization of Fiber Bragg Grating (FBG) and HC-PCF sensors.

The RI of a gas depends not only on gas species but also on ambient temperature and pressure. All the experiments were conducted at atmospheric pressure and room temperature. However, fluctuation of ~1 °C was recorded using an FBG sensor during the experiments, a result shown in Figure 6b. Therefore, it is required to characterize the temperature sensitivity of the HC-PCF MZI sensor before deploying the sensor for applications in the field. As part of the present research, HC-PCF sensors were placed in an oven, and the temperature was varied from 35 °C to 65 °C in 10 °C increments. Figure 10 displays the resulting correlation between recorded wavelength shifts and measured temperatures of the sensors and FBG. The temperature sensitivities of sensors A, B, and C were found to be 33.1 pm/°C, 31.6 pm/°C, and 20 pm/°C, respectively. This finding shows that the temperature sensitivity of the fiber-optic interferometer decreases when the length of the HC-PCF decreases. As shown in Figure 10, a typical FBG has a temperature sensitivity of 10 pm/°C and it is insensitive to ambient RI change. Therefore, an in-line or parallel FBG can be placed as a reference to eliminate temperature cross-sensitivity in ambient RI measurement for practical applications. Like temperature, a fiber-optic pressure gauge that is insensitive to ambient RI can be used to eliminate pressure cross-sensitivity in real-life measurement. 

## 5. Conclusions

A compact fiber-optic MZI sensor is proposed and has been experimentally demonstrated for ultra-high sensitive detection of gases. Different lengths of HC-PCF stubs were used to construct and characterize several sensors. The resulting MZI sensors were able to measure the RI of target gases and showed great sensitivity to measurand gases. The Refractive index sensitivity of 4629 nm/RIU was achieved for the MZI with an HC-PCF length of 3.30 mm. The RI sensitivity of the proposed MZI sensor inversely relates to the length of the HC-PCF stub. However, response and recovery times turned out to be shorter for longer HC-PCF stubs. The effect of gap distances on the number and amplitude distribution of the sensors’ modes was examined, and spatial frequency analysis revealed that power is mainly carried by two dominant modes in the proposed MZI. These novel and compact sensors have high-temperature sensitivity, compared to an FBG. With appropriate packaging, the proposed sensor becomes robust and is a suitable choice for low-percentage detection of gases as well as environmental monitoring. 

## Figures and Tables

**Table 1:** Transmission fringe shift of the MZI sensors for helium, methane, and argon.

Sensor	HC-PCF Length	Spectral Shift (pm) in Helium	Spectral Shift (pm) in Methane	Spectral Shift (pm) in Argon	RI Sensitivity (nm/RIU)
A	4.97 mm	780	440 (negative)	45	3019
B	4.73 mm	1060	600 (negative)	70	4300
C	3.30 mm	1300	618 (negative)	100	4629

**Table 2:** RI sensitivity comparison for gas sensing with other reported fiber-optic gas sensors.

Optical Structure	RI Range	RI Sensitivity (nm/RIU)	Reference
Proposed HC-PCF MZI	1.000034–1.000449	4629	This work
HC-PCF MZI	1.0000–1.0005	1233	[37]
Fabry-Perot (FP) based on hollow silica tube	1.00027–1.00189 1.00007–1.00051	1546	[48]
Surface plasmon resonance (SPR) with metallic surface grating (tapered SMF)	1–1.41	500	[49]
Hybrid optical fiber FP interferometer	1.0005–1.00275	560	[50]
SPR based on fiber grating in multi-mode fiber	1–1.33	280	[51]
Cavity based FP	1.0000–1.0025	1053	[52]
Open cavity MZI	1–1.02	3402	[26]

**Table 3:** Response and recovery times of HC-PCF MZI sensors to different gases.

HC-PCF Length (mm)	A (4.97)	B (4.73)	C (3.30)
Helium: response (s)/recovery (s)	50/50	50/55	57/57
Methane: response (s)/recovery (s)	32/39	44/46	46/56
Argon: response (s)/recovery (s)	37/44	62/49	110/100
